# Practical Points in Hospital Mechanics

**Published:** 1914-05-16

**Authors:** 


### PRACTICAL POINTS IN HOSPITAL MECHANICS.

(Criticism and Suggestions Invited.)

### The Control of Air Temperature by Thermostats.

The question of proper ventilation is being more and more closely studied for public buildings of all kinds, including the various departments of a hospital. As is well known, in some hospitals the "Plenum System" is adopted. In this, the air is cleaned, dried, moistened, cooled, warmed, or modified as desired, and delivered into the wards.

### The "Plenum System."

When the present writer made an investigation into the working of the "Plenum System," at the request of the Editor of this Journal some years ago, he found that doctors and nurses objected strongly to it in their quarters, and that doctors complained that convalescents did not get on.

The writer suggests now that the temperature and humidity in each ward, and particularly in operating theatres, might be controlled automatically by the nurse or sister. In operating theatres, it appears to him, it would be of very great service to have a control of this kind; and further to be able to admit a definite quantity of ozone as and when it was required.

In the Manchester School of Technology the control of the temperature in the different rooms of the building is obtained by the aid of a valve worked by hand. Two currents of air are led to each room, and by the motion of the valve the proportion of the two currents is changed. It is only a short step from this to controlling the temperature automatically by a thermostat.

There are a number of thermostats on the market; a large proportion of them depend upon the use of an electric current, which is put in operation when the temperature reaches a certain figure. The arrangements vary. Where rooms, or wards, are heated by hot water or steam radiators, an electro-magnet can be attached to each radiator, and operates a valve controlling the flow of hot water or steam through the radiator. The wires of the electro-magnet form part of a circuit, in which is included a thermometer fixed in a convenient part of the ward. In the thermometer is a platinum wire, sometimes sealed into the glass at a certain definite figure, sometimes arranged to be moved to and fro. Where the platinum wire is movable, it passes through a cork fixed in the top of the tube. In either case, when the temperature reaches the figure at which the platinum wire is set, the electric circuit is completed, the electro-magnet is energised, and the flow of hot water or steam is cut off. When the temperature in the neighbourhood of the thermometer falls, the mercury is supposed to recede from the wire, the circuit to be broken, the electro-magnet to release the valve, and the flow of hot water or steam to continue. In a variation of this, designed largely for dyeing-houses in the textile industries, the flow of steam is controlled by a specially balanced valve, the balance being upset when an electric circuit is closed, the contact being made in a thermometer in the manner described above. The difficulty which the writer has found in connection with a thermometer operating in this way is that the mercury is loth to leave the platinum. Modifications of the arrangement have been made, such as a globule of mercury falling over when a contact is to be made, and falling back when the contact is to be broken. This is better, providing it works properly. The electrical thermostat is also used to control the electric current flowing to electrical radiators or convectors. This arrangement is very simple; but it is open to the same objection. Contacts at a certain definite temperature are sometimes made by the unequal expansion of two metals; but the difficulty with this method is that there is not the room for regulation that exists with the mercury thermometer. By making the mercury thermometer large, and consequently increasing the weight of the column of mercury, the difficulty of breaking contact has been at least partly overcome.

Compressed air and volatile liquids are also used to work thermostats. With compressed air, a carefully balanced valve forms part of an air circuit, and is exposed to a definite pressure of air from a reservoir of a definite size. If the temperature rises, the pressure of the air rises, and the small movement of the valve is made to operate a mechanism controlling the supply of heat.

The apparatus in which a volatile liquid is employed has various forms. In all of them a small quantity of the liquid is held in a receptacle, one side of which is closed by a flexible diaphragm. When the temperature rises to a certain definite figure a portion of the liquid is formed into vapour, a pressure is brought to bear upon the diaphragm, and either an electric circuit is closed or some other mechanism is put in operation, shutting off the supply of the heating agent.

### The Use of Ozone.

The present writer, after his investigation of the "Plenum System," suggested, also in The Hospital, that ozone might be added to the air current, particularly for operating theatres. The method is actually in use in America and in this country, though not, so far as our information goes, for operating theatres. Ozone is made by an electrical apparatus designed for the purpose, and is delivered to the air current in certain definite quantities. It is found to be of great service in hotel dining-rooms, for instance, in drapers' work-rooms, etc. It is, therefore suggested that ozone could be delivered to the ventilating air current for operating theatres, and could be controlled by a simple hand switch, fixed in the operating theatre itself, the switch being worked by the sister in charge. Moreover, the strain of operations upon everyone concerned would be very much relieved by the aid of the ozone current.

